# Exercise: *primus inter pares* of Life’s Simple 7

**DOI:** 10.18632/aging.203041

**Published:** 2021-05-07

**Authors:** Amani M. Norling, Thomas W. Buford, Ronald M. Lazar

**Affiliations:** 1 The UAB Evelyn F. McKnight Brain Institute, Department of Neurology, University of Alabama at Birmingham, Birmingham, AL 35233, USA; 2 The University Center for Exercise Medicine, Department of Medicine, University of Alabama at Birmingham, Birmingham, AL 35233, USA

**Keywords:** aging, brain, CBF, cognition, exercise, IGF-1, Life’s Simple 7, vascular

Non-pathological brain aging is characterized by progressive deteriorations in brain structure and function, which predispose older individuals to age-related cognitive decline that impacts multiple functional domains and increases dementia risk. With increasing lifespan, there has been a corresponding rise in the prevalence of cognitive impairment and dementia resulting in a marked public health burden [1]. Once pathologically-based decline begins, few therapeutic options currently exist to halt or slow the downward course. The question therefore arises about what individuals can do to proactively mitigate or forestall the onset of decline before it happens.

**Figure 1:**
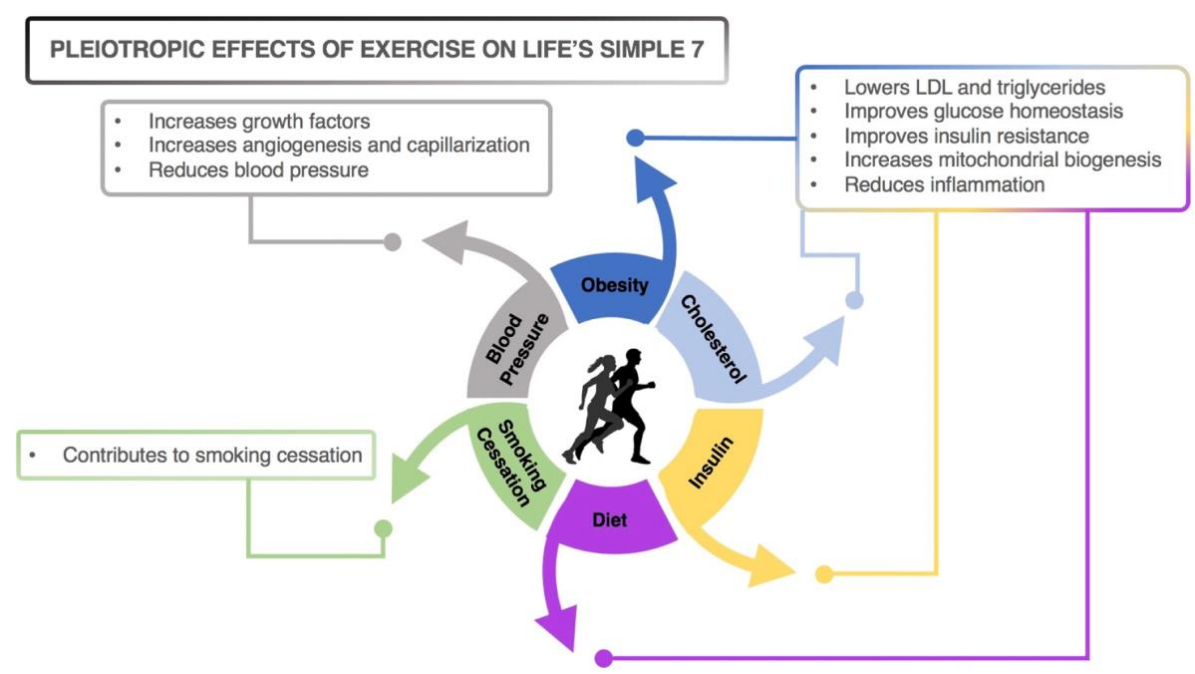
Pleiotropic effects of exercise on Life’s Simple 7.

In 2010, the American Heart Association/American Stroke Association (AHA/ASA) published an evidence-based Scientific Statement regarding the cardiovascular health benefits of meeting targets for seven risk factors and lifestyle behaviors (“Life’s Simple 7”) [2], which were later extended to optimal brain health and cognition [3]. These include: control of cholesterol and sugar levels; blood pressure management; smoking cessation, weight control, healthful diet, and exercise to increase cardiovascular and brain health. While the beneficial effects of each one of the AHA/ASA’s factors are important contributors to cardiovascular and brain health, we propose that exercise-induced cardiorespiratory fitness (CRF) stands in the forefront of the cardiovascular benefits and is *primus inter pares* “first among equals” of Life’s Simple 7 (see Figure 1).

A large body of evidence supports our premise: regular exercise, via its pleiotropic physiologic actions, is an effective anti-depressant, aids in weight control and smoking cessation [4], protects against the negative effects of suboptimal diets on cholesterol and atherogenesis, insulin resistance, and hypertension [5]. Importantly, studies show that high CRF is strongly associated with better cognitive function [1].

Although the physiological mechanisms by which exercise exerts its beneficial effects on the brain and cognition remain incompletely identified, several exercise-induced benefits are worthy of mention. Exercise/improvement in CRF increases brain volume and seems to induce a reduction in systemic and central inflammation [5]. It also stimulates neurogenesis via an increase in brain-derived neurotrophic factor (BDNF) in the dentate gyrus of the hippocampus– an area highly sensitive to reductions in blood flow and the effects of aging– improves age-related growth hormone deficiencies, enhances angiogenesis, and reverses age-related reductions in vascular density and cerebral blood flow [6]. Furthermore, a major effect of exercise and CRF is on the lowering of blood pressure. It is widely accepted that hypertension contributes to the formation of white matter hyperintensities, which have been strongly associated with ischemic stroke risk and cognitive decline. Recently, we presented converging evidence that linked vascular dysfunction that arises as a result of age-related decrease in growth hormone and insulin-like growth factor-1 (IGF-1) on microvascular rarefaction (MVR) and loss of blood vessels, leading to primary hypertension. Based on our review of the literature, we proposed that IGF-1 deficiencies reduce vascular density, and induce MVR [6], that leads to an increase in vascular resistance, hypertension, reductions in CBF, and ultimately cognitive decline. Given the large body of evidence from exercise studies that shows exercise-induced improvements in hypertension and cognition, we suggest the following pathway that may explain its benefits on the brain: muscular contractions during exercise initiate a growth hormone cascade via the paracrine pathway resulting in an increase in neurotrophic factors such as IGF-1, vascular endothelial growth factor (VEGF), and angiogenesis. The increase in blood vessel density reduces vascular resistance, and reverses hypertension. There is compelling evidence demonstrating that IGF-1 is a key facilitator of exercise-induced, growth-factor release including BDNF, such that blocking of IGF-1 inhibits the effects of BDNF on brain function in animals [6]. More recently, the role of exercise in vascular health and CBF was highlighted in a study that demonstrated that exercise-induced CRF was associated with improvements in CBF and cerebrovascular function and cognition in older adults [7]. These latter findings are especially meaningful following reports that reductions in CBF precede buildup of disease-inducing proteins and may predict transition to Alzheimer’s pathology [8].

In summary, the AHA/ASA’s recommendation of Life’s Simple 7 is a noteworthy step toward attenuation of cardiovascular diseases and brain dysfunction. Here, we underscore the importance of exercise as a potentiator of the benefits of the AHA/ASA’s 7 heath behaviors due to its broad effects on cardiovascular risk factors and brain health. Undoubtedly, further studies are needed to fill the gaps in our knowledge regarding how exercise benefits the brain, and we invite our colleagues, many of whom have made significant contributions to this field, to explore this area of research.
